# Computer-Aided Diagnosis of Gastrointestinal Protruded Lesions Using Wireless Capsule Endoscopy: A Systematic Review and Diagnostic Test Accuracy Meta-Analysis

**DOI:** 10.3390/jpm12040644

**Published:** 2022-04-17

**Authors:** Hye Jin Kim, Eun Jeong Gong, Chang Seok Bang, Jae Jun Lee, Ki Tae Suk, Gwang Ho Baik

**Affiliations:** 1 Department of Internal Medicine, Hallym University College of Medicine, Chuncheon 24253, Korea; khyejin1027@hanmail.net (H.J.K.); gong-eun@hanmail.net (E.J.G.); ktsuk@hallym.ac.kr (K.T.S.); baikgh@hallym.or.kr (G.H.B.); 2 Institute for Liver and Digestive Diseases, Hallym University, Chuncheon 24253, Korea; 3 Institute of New Frontier Research, Hallym University College of Medicine, Chuncheon 24253, Korea; iloveu59@hallym.or.kr; 4 Division of Big Data and Artificial Intelligence, Chuncheon Sacred Heart Hospital, Chuncheon 24253, Korea; 5 Department of Anesthesiology and Pain Medicine, Hallym University College of Medicine, Chuncheon 24253, Korea

**Keywords:** artificial intelligence, computer-aided diagnosis, capsule endoscopy, polyp, tumor, protruded, lesion capsule endoscopy, ulcer, hemorrhage

## Abstract

Background: Wireless capsule endoscopy allows the identification of small intestinal protruded lesions, such as polyps, tumors, or venous structures. However, reading wireless capsule endoscopy images or movies is time-consuming, and minute lesions are easy to miss. Computer-aided diagnosis (CAD) has been applied to improve the efficacy of the reading process of wireless capsule endoscopy images or movies. However, there are no studies that systematically determine the performance of CAD models in diagnosing gastrointestinal protruded lesions. Objective: The aim of this study was to evaluate the diagnostic performance of CAD models for gastrointestinal protruded lesions using wireless capsule endoscopic images. Methods: Core databases were searched for studies based on CAD models for the diagnosis of gastrointestinal protruded lesions using wireless capsule endoscopy, and data on diagnostic performance were presented. A systematic review and diagnostic test accuracy meta-analysis were performed. Results: Twelve studies were included. The pooled area under the curve, sensitivity, specificity, and diagnostic odds ratio of CAD models for the diagnosis of protruded lesions were 0.95 (95% confidence interval, 0.93–0.97), 0.89 (0.84–0.92), 0.91 (0.86–0.94), and 74 (43–126), respectively. Subgroup analyses showed robust results. Meta-regression found no source of heterogeneity. Publication bias was not detected. Conclusion: CAD models showed high performance for the optical diagnosis of gastrointestinal protruded lesions based on wireless capsule endoscopy.

## 1. Introduction

Wireless capsule endoscopy (WCE) is the primary choice for the examination of patients with suspected small intestinal lesions who showed negative radiologic examination results. With the technical advancements, such as optical assembly, battery, and sensor modules, WCE allows the non-invasive visualization of all gastrointestinal mucosa. It provides about 50,000 images in one examination, and there is a minimal risk for discomfort or procedure-related adverse events [1]. Despite these benefits in clinical practice, WCE has a limitation in terms of interpretation. A tedious reading time of >1 h is needed, and there is a risk of oversight because only a few abnormal video frames might appear in a single examination [1,2].

Computer-aided diagnosis (CAD) has been adopted for the immediate interpretation of images obtained from gastrointestinal endoscopy [3,4]. These models use machine learning-based algorithms or deep learning-based neural networks to find the local features in given images and to provide an established model optimization [5]. An automatic detection or classification of abnormal lesions on endoscopic images or movies has been widely investigated and has shown promising results [6,7,8,9]. The most beneficial point of the application of CAD models in clinical practice would be the reduction of the burden on endoscopists [10]. CAD models could reduce the laborious reading time due to the automatic detection and classification of gastrointestinal abnormalities. These can help in detecting hidden or hard-to-detect lesions in real time, leading to a reduced miss rate of important findings in WCE [11]. Another benefit would be its highly accurate diagnostic performance, which is comparable to that of an endoscopist [7,9]. These CAD models are expected to help in the automatic detection and diagnosis of important and hard-to-detect lesions using WCE images, making it possible to automatically read the entire WCE movies.

Protruded lesions in the gastrointestinal tract include various abnormalities, such as neoplasms, benign polyps, and other mucosal elevations (edematous ulcerations, venous structures, such as varix or bleb). Identifying and making an accurate diagnosis for these tumorous lesions are important, especially for lesions located in the small bowel, which is difficult to access through conventional endoscopy. Previous studies have reported the performance of each established CAD model in the diagnosis of protruded lesions using WCE images [12,13,14,15,16,17,18,19,20,21,22,23]. However, there are no studies that systematically determine the performance of CAD models in diagnosing gastrointestinal protruded lesions. The aim of this study was to evaluate the diagnostic performance of CAD models for gastrointestinal protruded lesions using wireless capsule endoscopic images.

## 2. Methods

### 2.1. Adherence to the Statement of Systematic Review and Diagnostic Test Accuracy Meta-Analysis

This study was conducted in accordance with the statement of the Preferred Reporting Items for a Systematic Review and Meta-analysis of diagnostic test accuracy (DTA) studies [24]. The protocol of this study was registered in the International Prospective Register of Systematic Reviews database before the initiation of the systematic review (ID 276623). The approval from the institutional review board of the Chuncheon Sacred Heart Hospital was waived.

### 2.2. Literature Searching Strategy

Searching formulas were made using keywords related to the performance of CAD models in diagnosing gastrointestinal protruded lesions using WCE images. Medical subject headings, terminologies, or author keywords were used to establish searching formulas (Table 1).

Two authors (C.S.B. and J.J.L.) independently conducted a database search of MEDLINE through PubMed, Web of Science, and Cochrane Library using the pre-established search formulas, from inception to August 2021. Duplicate articles were excluded. The titles and abstracts of all identified articles were reviewed, and irrelevant articles were excluded. Full-text reviews were subsequently conducted to determine whether the pre-established inclusion criteria were satisfied in the identified studies. The references to relevant studies were also reviewed to identify any additional articles. Any disagreements in the results obtained from the searching process between the two authors were resolved by discussion or consultation with a third author (G.H.B.).

**Table 1:** Literature searching strategy.

**Database: MEDLINE (through PubMed)**
#1 “artificial intelligence”[tiab] OR “AI”[tiab] OR “deep learning”[tiab] OR “machine learning”[tiab] OR “computer”[tiab] OR “neural network”[tiab] OR “CNN”[tiab] OR “automatic”[tiab] OR “automated”[tiab]: 536153 #2 “capsule endoscopy”[tiab] OR “capsule endoscopy”[Mesh]: 5136 #3 “protruded”[tiab] OR “polyp”[tiab] OR “tumor”[tiab] OR “tumors”[Mesh] OR “polyps”[Mesh]: 1295552 #4 #1 AND #2 AND #3: 52 #5 #4 AND English[Lang]: 51
**Database: Web of Science**
#1 artificial intelligence OR AI OR deep learning OR machine learning OR computer OR neural network OR CNN OR automatic OR automated: 130090 #2 capsule endoscopy: 3549 #3 protruded OR polyp OR tumor: 840061 #3 #1 AND #2 AND #3: 110
**Database: Cochrane Library**
#1 artificial intelligence:ab,ti,kw or AI:ab,ti,kw or deep learning:ab,ti,kw or machine learning:ab,ti,kw or computer:ab,ti,kw or neural network:ab,ti,kw or CNN:ab,ti,kw or automatic:ab,ti,kw or automated:ab,ti,kw: 60782 #2 MeSH descriptor: [capsule endoscopy] explode all trees: 132 #3 capsule endoscopy:ab,ti,kw: 726 #4 #2 or #3: 726 #5 MeSH descriptor: [tumors] explode all trees: 83592 #6 MeSH descriptor: [polyps] explode all trees: 1165 #7 protruded:ab,ti,kw or tumor:ab,ti,kw or polyp:ab,ti,kw: #8 #5 or #6 or #7: 134070 #9 #1 and #4 and #8: 5

CAD, computer-aided diagnosis; WCE, wireless capsule endoscopy; tiab, searching code for title and abstract; Mesh, Medical Subject Headings; ab,ti,kw, searching code for abstract, title, and keywords; Lang, searching code for language; lim, searching code by limiting certain conditions.

### 2.3. Inclusion Criteria

The studies included in this systematic review met the following inclusion criteria: studies designed to evaluate the diagnostic performance of CAD models for gastrointestinal protruded lesions based on WCE images; studies that presented the diagnostic performance of CAD models, including sensitivity, specificity, likelihood ratios, predictive values, or accuracy, which enabled the estimation of true positive (TP), false positive (FP), false negative (FN), and true negative (TN) values of CAD models; and studies written in English. The exclusion criteria were as follows: narrative review articles, studies with incomplete data, systematic review, or meta-analyses, proceedings with abstract only, and study protocols. Full publications with PDF files of available proceedings were considered full articles. Articles that met at least one of the exclusion criteria were excluded from this study.

### 2.4. Methodological Quality

The methodological quality of the included studies was assessed by two authors (C.S.B. and J.J.L.) using the second version of Quality Assessment of Diagnostic Accuracy Studies (QUADAS-2). This tool comprised four domains, namely “patient selection,” “index test,” “reference standard,” and “flow and timing,” and the first three domains have an “applicability” assessment. The two authors (C.S.B. and J.J.L.) evaluated each part as having either a high, low, or unclear risk of bias, and any disagreements in the results in the searching process between the two authors were resolved by discussion or consultation with a third author (G.H.B.) [25].

### 2.5. Data Extraction, Primary Outcomes, and Additional Analyses

Two authors (C.S.B. and J.J.L.) independently extracted the data from each included study, and the extracted data were cross-checked. If data were unclear, the corresponding author of the study was contacted by e-mail. A descriptive synthesis was done through a systematic review process, and a DTA meta-analysis was done if the included studies were sufficiently homogenous.

The primary outcomes were the TP, FP, FN, and TN values of each study. For the CAD of gastrointestinal protruded lesions using WCE images, the primary outcomes were defined as follows: TP, the number of subjects with a positive finding on a CAD model and with protruded lesions based on WCE images; FP, the number of subjects with a positive finding on a CAD model and with no protruded lesions based on WCE images; FN, the number of subjects with a negative finding on a CAD model and with protruded lesions based on WCE images; and TN, the number of subjects with a negative finding on a CAD model and with no protruded lesions based on WCE images. With these definitions, TP, FP, FN, and TN values were calculated for each included study.

For additional analyses of meta-regression and subgroup analysis, the following variables were extracted from each included study: published year, geographic origin of the data (i.e., Asian vs. Western vs. Publica data or Unknown), type of CAD models, number of total images included in the training datasets and test datasets, type of test datasets (internal test vs. external test), and target conditions (polyps vs. tumors vs. other protruded lesions).

### 2.6. Statistical Analysis

The hierarchical summary receiver operating characteristic (HSROC) method was primarily adopted for the DTA meta-analysis [26]. A forest plot of the pooled sensitivity or specificity and a summary ROC (SROC) curve were also generated. The level of heterogeneity across the included articles was determined based on correlation coefficients between the logit-transformed sensitivity and specificity using the bivariate method [27] and asymmetry parameter β, where β = 0 corresponds to a symmetric ROC curve in which the diagnostic odds ratio (DOR) does not vary along the curve according to the HSROC method [26]. A positive correlation coefficient and a β with a significant probability (*p* < 0.05) indicate heterogeneity between studies [26,28]. A visual inspection of the SROC curve was also done to identify the heterogeneity. Subgroup analysis by univariate meta-regression using the modifiers identified during the systematic review was also conducted to identify the reasons for heterogeneity. The METANDI and MIDAS packages in the STATA software version 15.1 (College Station, TX, USA) were used for the DTA meta-analysis. Deeks’ funnel plot asymmetry test was conducted to determine the publication biases. For the subgroup analyses of less than four studies, the Moses-Shapiro-Littenberg method [29] was used in the Meta-DiSc 1.4 (XI Cochrane Colloquium, Barcelona, Spain) software because the METANDI and MIDAS packages in the STATA software require the inclusion of a minimum of four studies for DTA meta-analysis.

## 3. Results

### 3.1. Study Selection Process

**Figure 1:**
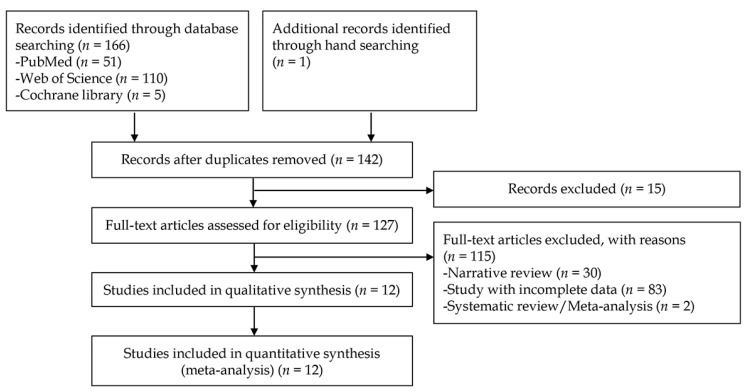
Flow chart of the study selection process for the diagnostic performance of computer-aided diagnosis for gastrointestinal protruded lesions using wireless capsule endoscopy.

A total of 167 studies were identified from the literature searching process on the three databases. One study was additionally identified by manual screening of references. After excluding duplicate studies, additional articles were excluded after reviewing their titles and abstracts. Full-text versions of the remaining 127 studies were obtained and thoroughly reviewed based on the aforementioned inclusion and exclusion criteria. Among these, 115 articles were excluded because these articles did not present the exact number of test images used in each study or simply presented one or two diagnostic performance outcomes. Therefore, the crude value of TP, FP, FN, and TN cannot be measured in the excluded studies. Finally, 12 studies [12,13,14,15,16,17,18,19,20,21,22,23] for the CAD of gastrointestinal protruded lesions were included in the systematic review. A flow chart of the study selection process is shown in Figure 1.

### 3.2. Clinical Characteristics

Among the 12 studies [12,13,14,15,16,17,18,19,20,21,22,23] for the CAD of gastrointestinal protruded lesions using WCE, a total of 28,148 images were identified (10,649 cases vs. 17,499 controls) for the assessment of the diagnostic performance. Six studies [15,16,18,19,22,23] used endoscopic images from Asian populations, and three studies [14,17,20] used endoscopic images from Western populations. However, three studies [12,13,21] used public database images searched on the internet or from unknown sources. In terms of the type of CAD model, a deep neural network or convolutional neural network was used in four studies [12,17,20,22], while machine learning-based models were used in eight studies [13,14,15,16,18,19,21,23]. In the context of the target lesions, seven studies [15,16,17,18,19,21,23] presented the diagnostic performance of the CAD of intestinal tumors, and four studies [12,13,14,20] presented its diagnostic performance for intestinal polyps. However, the study by Saito H et al. (2020) [22] presented an indistinguishable performance of the CAD of protruded lesions. Therefore, subgroup analyses were performed for the target lesions. The detailed clinical features of the included studies are presented in Table 2.

**Table 2:** Clinical characteristics of the included studies for the diagnosis of gastrointestinal protruded lesions in wireless capsule endoscopy images using computer-aided diagnosis.

Study/Year	Nationality of Data	Type of CAD Models	Type of Endoscopic Images	Training Dataset	Type of Test Datasets	Number of Protruded Lesions in Test Dataset	Number of Controls in Test Dataset	TP	FP	FN	TN	Target Conditions
Li B et al. (2009) [12]	unknown	Feature analysis (texture, color) with MLP	Still cut images	150 polyp images and 150 normal mucosal images	Internal test	150	150	134	6	16	124	for small bowel polyp diagnosis
Hwang S (2011) [13]	unknown	BoW model-SVM	Still cut images	25 polyp images and 50 normal mucosal images	Internal test	50	100	33	5	17	95	For small bowel polyp diagnosis
Karargyris A et al. (2011) [14]	US	Texture analysis with SVM	Still cut images	unclear	Internal test	10	40	10	13	0	27	For small bowel polyp diagnosis
Li B et al. (2011) [15]	China	Texture analysis with SVM	Still cut images	550 tumor images and 550 normal mucosal images	Internal test	50	50	45	1	5	49	for small bowel tumor diagnosis
Li B et al. (2011) [16]	China	Texture analysis with an ensemble of kNN, MLP, or SVM	Still cut images	450 tumor images and 450 normal mucosal images	Internal test	150	150	138	17	12	133	for small bowel tumor diagnosis
Barbosa DC et al. (2012) [17]	Portugal	Texture analysis with neural network	Still cut images	700 tumor images and 2300 normal mucosal images	Internal test	700	2300	657	159	43	2141	for small bowel tumor diagnosis
Li B et al. (2012) [18]	China	Texture analysis with SVM	Stil lcut images	540 tumor images and 540 normal mucosal images	Internal test	60	60	51	11	9	49	for small bowel tumor diagnosis
Li B et al. (2012) [19]	China	Texture analysis with SVM	Still cut images	540 tumor images and 540 normal mucosal images	Internal test	60	60	53	2	7	58	for small bowel tumor diagnosis
Constantinescu AF et al. (2015) [20]	Romania	Texture analysis with neural network	Still cut images	unclear	Internal test	32	58	30	5	2	53	for intestinal polyp diagnosis
Kundu AK et al. (2020) [21]	from http://www.capsuleendoscopy.org	Linear discriminant analysis with SVM	Still cut images	30 tumor images and 1617 normal mucosal images	Internal test	30	1617	26	130	4	1487	for small bowel tumor diagnosis
Saito H et al. (2020) [22]	Japan	CNN	Still cut images	30,584 images of protruding lesions	Internal test	7507	10000	6810	2019	697	7981	for protruding lesion diagnosis (small bowel)
Yamada A et al. (2020) [23]	Japan	Single Shot MultiBox Detector	Still cut images	15933 images	Internal test	1850	2934	1462	380	388	2554	for colorectal tumor diagnosis

CAD, computer-aided diagnosis; TP, true positive; FP, false positive; FN, false negative; TN, true negative; MLP, multilayer perceptron; BoW, Bag-of-Words; SVM, support vector machine; RFE, recursive feature elimination.

### 3.3. Methodological Quality Assessment

CAD models were established based on the input training data. Therefore, the quality and quantity of the baseline training data are important. A sufficient number of training images that have various important features are required to establish practical models. Endoscopists should also participate in the labeling work for accurate preparation of the training data. If the established CAD models used the training images from public databases searched on the internet, the quality of these training data cannot be guaranteed.

The authors defined that proper learning requires at least 30 training images (quantity standard) from real clinic hospital data (quality standard) labeled by endoscopists (quality standard). If both the quality and quantity standards were satisfied, it was defined as a low risk of bias in the patient selection domain. If only one of these quality or quantity standards was satisfied, it was defined as unclear risk of bias. If both were not satisfied, it was defined as a high risk of bias.

**Figure 2:**
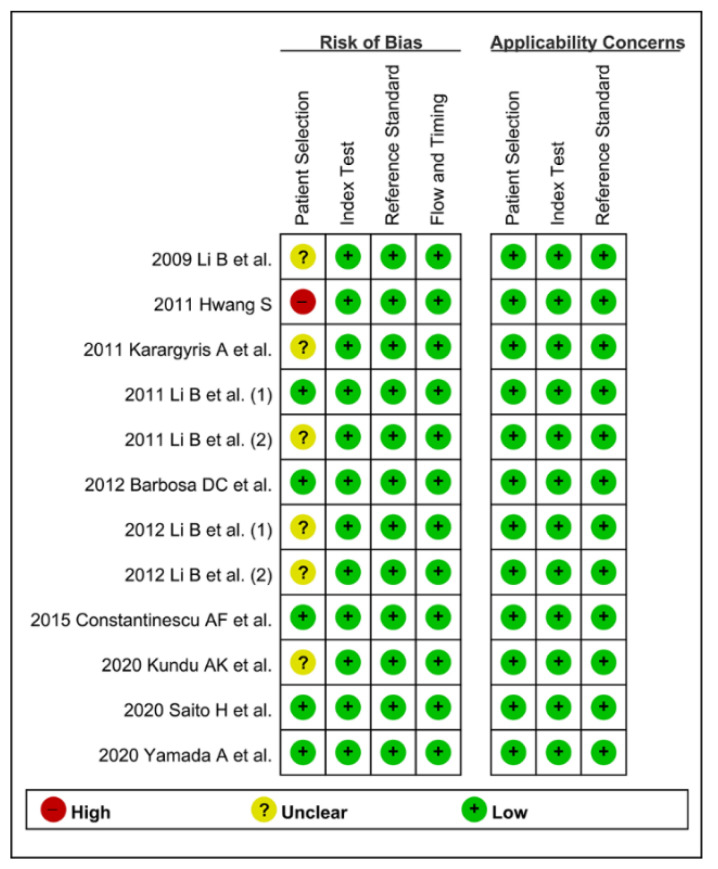
Summary graph of quality in methodology for the computer-aided diagnosis of gastrointestinal protruded lesions in wireless capsule endoscopy. (+) denotes low risk of bias, (?) denotes unclear risk of bias, (−) denotes a high risk of bias.

For the methodological quality assessment using QUADAS-2, only five studies [15,17,20,22,23] were rated with a low risk of bias, six studies [12,14,16,18,19,21] were rated with an unclear risk of bias, and one study [13] was rated with a high risk of bias in the patient selection domain. The remaining domains were rated with a low risk of bias in all the included studies (Figure 2). Therefore, the classification of methodological quality in the patient selection domain was adopted as a modifier in the subgroup or meta-regression analysis.

### 3.4. DTA Meta-Analysis

**Figure 3:**
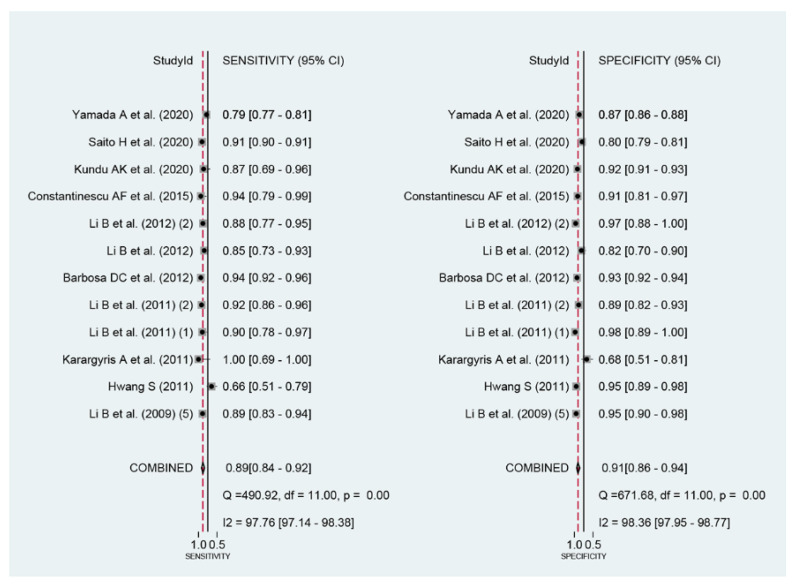
Coupled forest plots of the sensitivity and specificity in the computer-aided diagnosis models for the diagnosis of gastrointestinal protruded lesions in wireless capsule endoscopy images.

**Figure 4:**
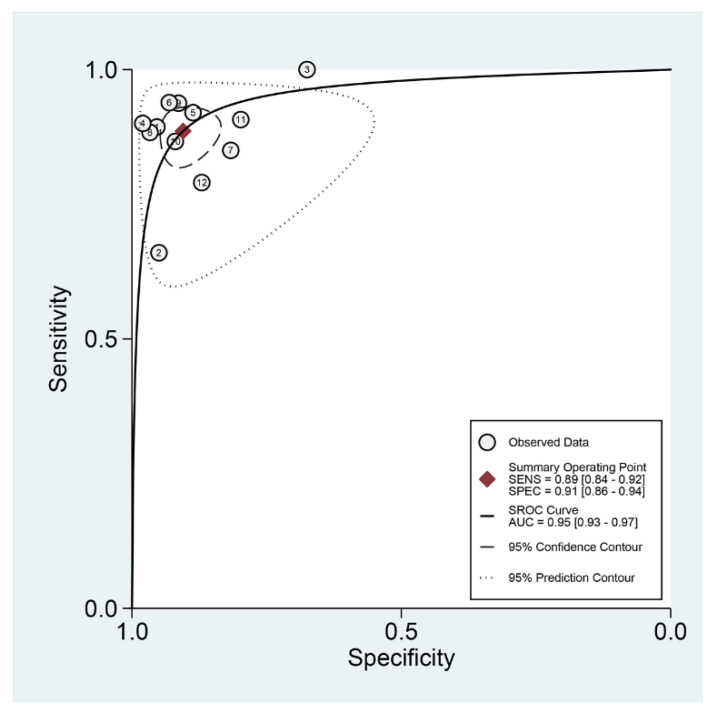
Summary receiver operating characteristic curve with 95% confidence region and prediction region of computer-aided diagnosis models for the diagnosis of gastrointestinal protruded lesions in wireless capsule endoscopy images.

**Figure 5:**
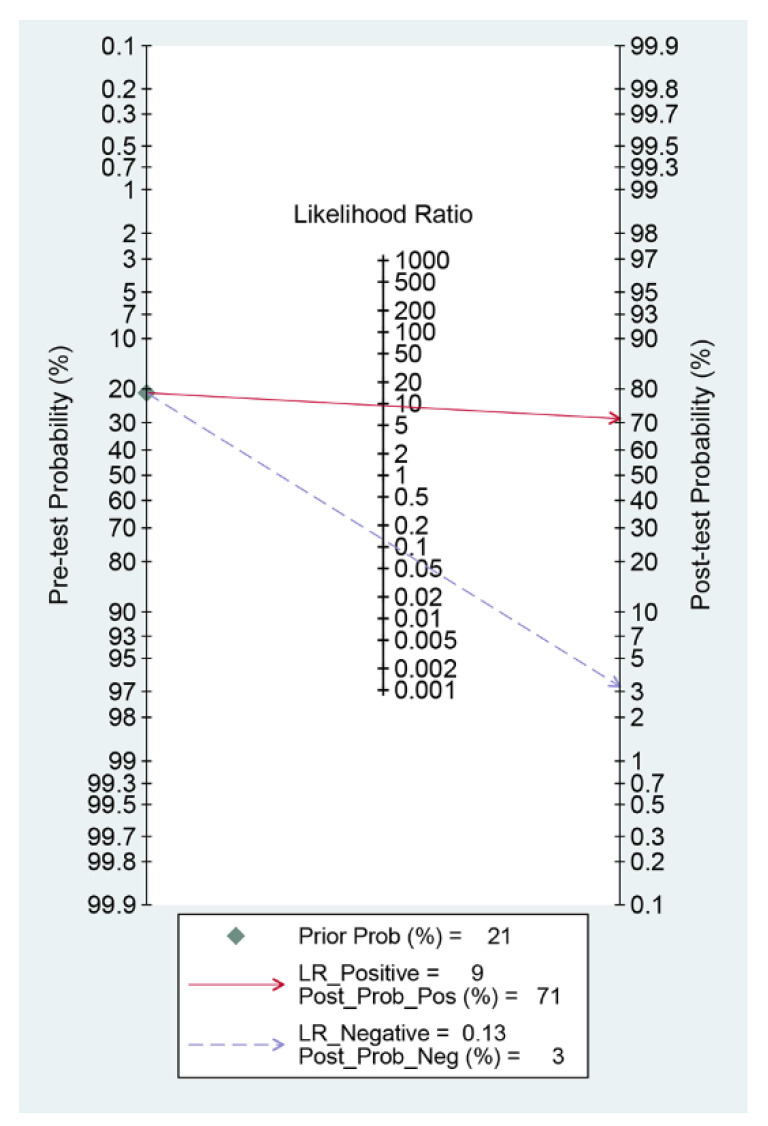
Fagan nomogram for the computer-aided diagnosis of gastrointestinal protruded lesions using wireless capsule endoscopy images.

Among the 12 studies [12,13,14,15,16,17,18,19,20,21,22,23] for the meta-analysis of the CAD of protruded lesions using WCE, the area under the curve, sensitivity, specificity, positive likelihood ratio, negative likelihood ratio, and DOR were 0.95 (95% confidence interval, 0.93–0.97), 0.89 (0.84–0.92), 0.91 (0.86–0.94), 9.3 (6.3–13.8), 0.13 (0.09–0.18), and 74 (43–126), respectively (Table 3, Figure 3). An SROC curve is shown in Figure 4. To investigate the clinical utility of the CAD models, a Fagan nomogram was generated. Positive findings indicate that gastrointestinal protruded lesions were detected by the CAD models, while negative findings indicate that gastrointestinal protruded lesions were not detected. Assuming a 21% prevalence of gastrointestinal protruded lesions based on WCE [30], the Fagan nomogram shows that the posterior probability of ulcers or erosions was 71% if the finding of the CAD model was positive and only 3% if the finding of the CAD model was negative (Figure 5).

### 3.5. Heterogeneity Evaluation, Meta-Regression, and Subgroup Analysis

First, a negative correlation coefficient between the logit-transformed sensitivity and specificity (r = −0.18) in the bivariate model analysis was observed, and an asymmetric β parameter in the HSROC model showed an insignificant *p-*value (*p* = 0.57), suggesting that heterogeneity does not exist in the included studies. Second, the study by Hwang (2011) [13] showed a lower sensitivity, and the study by Karargyris et al. (2011) [14] showed a lower specificity compared with the enrolled studies in a coupled forest plot of sensitivity and specificity (Figure 3). These studies have a high risk of bias and an unclear risk of bias in the methodology quality assessment, respectively (Figure 2). Therefore, a subgroup analysis was conducted according to the methodological quality, and the performance was robust; however, slightly higher values were observed in studies with high methodological quality (Table 3). Third, the shape of the SROC curve was symmetric, and the 95% prediction region was not wide, suggesting that there was no heterogeneity between the included studies (Figure 4). Fourth, a meta-regression using the modifiers identified in the systematic review was conducted, with no source of heterogeneity found (published year [*p* = 0.28], number of test images [*p* = 0.33], type of CAD models [*p* = 0.17], and target disease (polyps vs. tumors vs. other protruded lesions) [*p* = 0.47]). Finally, a subgroup analysis based on the potential modifiers was performed, and the overall performance of the studies showed robust results (Table 3).

**Table 3:** Summary of performance and subgroup analysis of the included studies for the diagnosis of protruded lesions in wireless capsule endoscopy images using computer-aided diagnosis.

Subgroup	Number of Included Studies	Sensitivity (95% CI)	Specificity (95% CI)	PLR	NLR	DOR	AUC
All the included studies	12	0.89 (0.84–0.92)	0.91 (0.86–0.94)	9.3 (6.3–13.8)	0.13 (0.09–0.18)	74 (43–126)	0.95 (0.93–0.97)
Ethnicity of data							
Asian	7	0.88 (0.83–0.91)	0.90 (0.84–0.93)	8.4 (5.4–13.2)	0.14 (0.10–0.19)	62 (33–117)	0.94 (0.92–0.96)
Public database or unknown ethnicity	2	0.84 (0.78–0.88)	0.95 (0.92–0.98)	16.3 (9.1–29.3)	0.20 (0.06–0.65)	81 (18–370)	Null
Western Published year	3	0.94 (0.92–0.96)	0.93 (0.92–0.94)	7.5 (2.4–23.2)	0.07 (0.05–0.09)	199 (142–280)	0.98 (0.97–0.99)
<10 years (published within 10 years)	7	0.89 (0.84–0.93)	0.89 (0.85–0.93)	8.5 (5.8–12.4)	0.12 (0.08–0.18)	70 (36–135)	0.95 (0.93–0.97)
>10 years	5	0.91 (0.77–0.97)	0.92 (0.82–0.96)	10.9 (5.3–22.4)	0.10 (0.04–0.25)	107 (54–210)	0.96 (0.94–0.98)
Total number of included images for the training dataset							
100≤	9	0.89 (0.86–0.92)	0.91 (0.87–0.94)	9.8 (6.5–14.6)	0.12 (0.09–0.16)	83 (46–151)	0.95 (0.93–0.97)
<100 or unknown	3	0.79 (0.70–0.87)	0.88 (0.83–0.93)	7.2 (1.9–26.9)	0.15 (0.03–0.71)	55 (23–134)	0.95 (0.91–0.99)
Total number of included images for the test dataset							
100≤	11	0.88 (0.84–0.92)	0.91 (0.88–0.94)	10.2 (7.1–14.6)	0.13 (0.09–0.18)	79 (46–134)	0.96 (0.93–0.97)
<100	1	Null	Null	Null	Null	Null	Null
Methodological quality of included studies							
High-quality	5	0.90 (0.84–0.94)	0.90 (0.84–0.94)	9.2 (5.3–15.8)	0.11 (0.07–0.18)	84 (34–208)	0.96 (0.94–0.97)
Unclear or low-quality	7	0.88 (0.80–0.93)	0.91 (0.84–0.95)	9.4 (5.6–15.9)	0.13 (0.08–0.21)	72 (39–131)	0.95 (0.93–0.97)
Type of CAD models							
Neural network-based	4	0.92 (0.91–0.94)	0.91 (0.84–0.95)	9.7 (5.5–17.3)	0.08 (0.06–0.11)	116 (53–254)	0.95 (0.93–0.97)
Machine learning-based	8	0.86 (0.79–0.91)	0.90 (0.84–0.94)	8.8 (5.3–14.5)	0.16 (0.10–0.23)	57 (30–108)	0.94 (0.92–0.96)
Type of target lesions							
Tumors	7	0.89 (0.85–0.93)	0.91 (0.89–0.93)	10.0 (7.8–12.7)	0.12 (0.08–0.17)	85 (46–156)	0.95 (0.93–0.97)
Polyps	4	0.94 (0.68–0.99)	0.91 (0.79–0.96)	10.3 (4.6–23.0)	0.07 (0.01–0.39)	148 (40–548)	0.97 (0.95–0.98)
Other protruded lesion	1	Null	Null	Null	Null	Null	Null

CI, confidence interval; PLR, positive likelihood ratio; NLR, negative likelihood ratio; DOR, diagnostic odds ratio; AUC, area under the curve; CAD, computer-aided diagnosis.

### 3.6. Publication Bias

**Figure 6:**
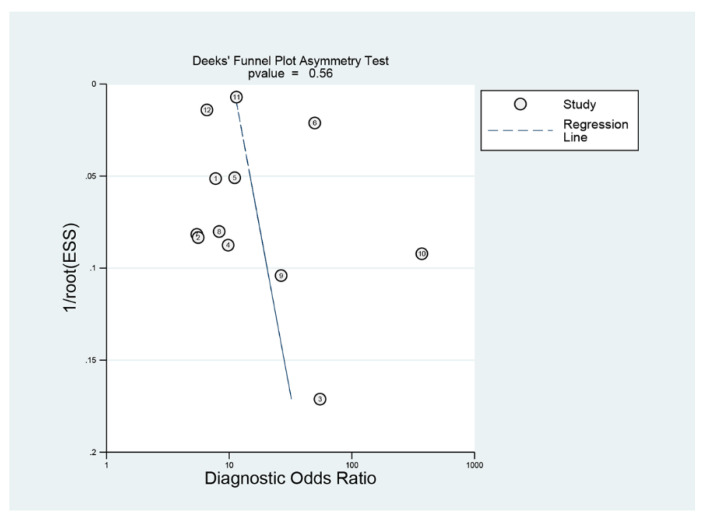
Deek’s funnel plot of computer-aided diagnosis models for the diagnosis of gastrointestinal protruded lesions in wireless capsule endoscopy images.

Deeks’ funnel plot showed a symmetrical shape with respect to the regression line (Figure 6), and the asymmetry test showed no evidence of publication bias (*p* = 0.56).

## 4. Discussion

### 4.1. Main Findings

The CAD models showed high pooled performance values for the diagnosis of gastrointestinal protruded lesions based on WCE images. Practical values in Fagan’s nomogram indicated the potential benefit of the CAD models in clinical practice. The meta-regression analysis showed no sources of heterogeneity, and subgroup analyses demonstrated a robust quality of evidence. Its diagnostic performance was high, regardless of whether the protruded lesions were tumors or polyps.

WCE has revolutionized the screening or diagnosis of gastrointestinal protruded lesions. It has been the diagnostic choice for patients with obscure gastrointestinal hemorrhage. With the advancement of optical technology and its own advantages, such as no risk of pain, air insufflation, or sedation, its indications are expanding. In addition to small bowel lesions, the applications of WCE for gastric or colonic lesions are being studied [31,32]. The lack of mobility, a disadvantage that has been raised for a long time despite its advantages, is being overcome by magnetic control or robotic capsule endoscopy [33,34].

Despite the benefits and advancement of WCE, its interpretation is still a tedious task for endoscopists.

Considerable time and focused attention are required for the interpretation of the whole images and movies of WCE. Therefore, there is a risk of oversight for important images [1,2]. A suspected blood indicator using color or texture analysis has been studied and used to improve the efficiency of interpretation [35,36]. Blood has a color that can be easily distinguished from surrounding intestinal mucosa, but protruded lesions are often similar in color and texture to the surrounding mucosa, making it difficult to distinguish them.

CAD using machine learning or deep learning is suitable for fields that are either too complex for conventional analysis methods or have no well-known rules. Combining a big data analysis with CAD can potentially increase its accuracy because analyzing large amounts of data can uncover unexpected associations or new trends. In this context, the CAD models in each study showed a high performance, and this was robust when either machine learning or deep learning was used to establish the CAD models. However, neural network-based CAD models showed a slightly higher performance than traditional machine learning-based CAD models (Table 3). This is presumed that image analysis with local feature extraction can be highly optimized with its complex layers, deep node calculations, and dimensional reductions for neural network-based CAD models [3,5]. Considering that the machine learning-based models in the included studies used color or texture features, neural network-based models might focus on other local features or combined features, such as the shape of the lesions or feature differences between the lesions and surrounding mucosa. Explainable artificial intelligence analyses are being studied, and the wide application of this analysis would reveal a discrete way of determination in the CAD models [37].

### 4.2. Limitations

Several inevitable limitations were identified during the systematic review process. First, experimental CAD models rather than practical models were established and studied. All the performance metrics in the included studies were measured in an internal test setting. Because a hypothesis was made in the model establishment, stating that observations fit certain statistical rules, external validation could confirm whether this hypothesis is expandable or generalizable. Therefore, the confirmation of the performance of the established models with new data is essential [9]. However, a practical model establishment with external validation was not conducted in all the included studies. Second, the number of studies with neural network-based model establishment [12,17,20,22] was lower than that of studies with machine learning-based model establishment [13,14,15,16,18,19,21,23]. Neural network-based models do not always have a better performance than machine learning-based models in all fields. However, considering that neural network-based models are being widely studied and the performance of the subgroup analysis showed slightly higher values in the neural network-based models than those in the machine learning-based models, the inclusion of more studies with neural network-based models would give new implications for this topic. Third, the utilized images were retrieved from a single institution [14,15,16,17,18,19,20,22,23]. Moreover, three studies [12,13,21] used public database images searched on the internet or from unknown sources. Due to the unique characteristics of patients in each institution, the CAD models developed from a single institution usually have limitations for widespread implementation, and the quality of the training data based only on internet searching cannot be guaranteed [9]. Fourth, nine of the twelve studies were published more than five years ago. Although the sensitivity analysis with the publication year produced robust results, it is necessary to re-evaluate the main outcomes, including future studies. Overall, training data with guaranteed quality and a balanced number of CAD model types that focus on external test-oriented performance are required and expected for future perspectives on this topic.

In conclusion, the CAD models showed high performance for the optical diagnosis of gastrointestinal protruded lesions in WCE.

## Data Availability

Not applicable.

## Abbreviations

CAD: computer-aided diagnosis; WCE, Wireless capsule endoscopy\; DTA: diagnostic test accuracy; TP: true positive; FP: false positive, FN: false negative, TN: true negative, QUADAS-2, the second version of Quality Assessment of Diagnostic Accuracy Studies: HSROC: hierarchical summary receiver operating characteristic SROC: summary receiver operating characteristicDOR: diagnostic odds ratio CI, confidence interval; PLR, positive likelihood ratio; NLR, negative likelihood ratio; DOR, diagnostic odds ratio; AUC, area under the curve; CAD, computer-aided diagnosis.
